# Promoting physical activity in primary care: a systematic review and meta-analysis

**DOI:** 10.3399/BJGP.2020.0817

**Published:** 2021-04-07

**Authors:** Veronika van der Wardt, Claudio di Lorito, Annika Viniol

**Affiliations:** Department of Primary Care, Philipps-Universität Marburg, Marburg, Germany.; Division of Rehabilitation, Ageing and Wellbeing, Queen’s Medical Centre, University of Nottingham, Nottingham, UK.; Department of Primary Care, Philipps-Universität Marburg, Marburg, Germany.

**Keywords:** behaviour change, family practice, motivation, physical activity, primary health care

## Abstract

**Background:**

Promoting physical activity is an important part of patient care in primary care and has been investigated in many studies with a wide range of intervention characteristics, often including external support. It is unclear, however, if promoting physical activity is effective.

**Aim:**

To investigate the effectiveness of behaviour change interventions to promote physical activity in primary care.

**Design and setting:**

This is a systematic review and meta-analysis to evaluate physical activity promotion in a primary care setting.

**Method:**

EMBASE, MEDLINE, PsycInfo, and the Joanna Briggs Institute Database were searched for ‘physical activity’, ‘interview’, ‘motivation’, ‘primary care’, and equivalent words to identify randomised controlled trials with physical activity as the outcome at patient level.

**Results:**

The review identified 25 eligible studies. The quality appraisal showed that most studies reported insufficient details regarding randomisation, group allocation, blinding, and fidelity of intervention delivery. The included studies reported a wide range of interventions with varying numbers of follow-up visits or phone calls. The overall effect size for interventions with a 6-month follow-up interval was 0.04 (95% confidence interval [CI] = −0.06 to 0.14), and for interventions with a 12-month follow-up interval it was 0.19 (95% CI = 0.03 to 0.36). Only one intervention based on three motivational interviewing sessions achieved a moderate effect.

**Conclusion:**

Counselling to promote physical activity in primary care has a limited effect on patients’ behaviour and it might not, on its own, be enough to change physical activity behaviour.

## INTRODUCTION

Exercise and physical activity (PA) reduce the risk of cardio- and cerebrovascular disease, cancer, obesity, and falls, and improve mental health, osteoporosis, and diabetes.^1^ The evidence for multiple benefits is strong, and shows that PA is key for healthy ageing.

Physical activity is defined as *‘any bodily movement produced by skeletal muscles that results in energy expenditure’*.^2^ Exercise is a particular type of PA, defined as *‘physical activity that is planned, structured, repetitive, and purposive in the sense that improvement or maintenance of one or more components of physical fitness is an objective’*.^2^

Current PA guidelines^3^ recommend that adults do at least 150–300 min of moderate intensity or 75–150 min of high-intensity aerobic PA per week, preferably spread throughout the week. In addition, adults should complete muscle-strengthening activities at least twice a week, and avoid sedentary behaviour for long, uninterrupted periods.

However, only about 30% of adults are sufficiently physically active in Europe, with figures ranging from 23% in Sweden to 44% in the Netherlands.^4^ Physical inactivity is associated with considerable costs for healthcare systems, particularly in high-income countries.^5^ These costs are predicted to increase over the next decades because of the ageing of the population.^6^

Primary care physicians are often the first point of contact for people to discuss their health. A consultation may present a suitable opportunity for patients to discuss PA levels, as this falls within the remit of primary care physicians.^7^^,^^8^ A systematic literature review showed that barriers to PA counselling included lack of incentives for the primary care physicians, time constraints, the perception of insufficient knowledge and training, and the lack of a counselling protocol on behaviour change.^8^ A survey indicated that about 75% of primary care physicians found it difficult to provide lifestyle modification counselling.^7^

At present, it is unclear which behaviour change strategies and support mechanisms primary care physicians should use to promote PA in their counselling sessions. There is a broad spectrum of behaviour change techniques, with at least 93 techniques available.^9^ A meta-analysis of 43 randomised controlled trials (RCTs) investigating behaviour change techniques for weight management and PA across settings showed that goal setting and self-monitoring were positively associated with intervention effect in the short and long term; exploring pros and cons of behaviour change produced inverse effects.^10^ In addition, giving feedback, setting graded tasks, and adding objects to the environment (such as a diet logbook) were associated with positive long-term effects.^10^ The study did not find any differences in effects when comparing different settings or weight management with PA.

In relation to primary care specifically, Noordman *et al* showed that a wide range of behavioural counselling interventions were effective.^11^ In addition, a health economics analysis indicated that most PA interventions set in primary care were cost-effective.^12^ However, both studies included interventions with characteristics that are not usually available in a primary care context (such as exercise coaches, health advisors, and physiotherapy programmes). Therefore, it remains unclear which interventions would be successful in supporting PA engagement when delivered in primary care settings. This systematic review of the literature and meta-analysis aimed to investigate interventions to promote PA that were delivered within a primary care context to evaluate their effectiveness. The research objectives were:

- to identify the types of behaviour change interventions that take place in primary care practices to support engagement in physical activities;
- to evaluate the effectiveness of behaviour change interventions delivered in a primary care context; and
- to determine which type of intervention is associated with moderate or large effect sizes.

**Table:** How this fits in

Though there is evidence that behaviour change promotion can have a positive effect when implemented across different settings, it is unclear how successful these interventions are when delivered in primary care without links to other support components (such as exercise classes). This systematic review and meta-analysis investigated physical activity promotion interventions exclusively delivered in primary care. Results indicated that interventions delivered by primary care providers only are unlikely to be sufficient and might need to be part of a comprehensive support system to successfully change behaviour.

## METHOD

The protocol for this systematic literature review was published on PROSPERO (CRD42020154879). Searches were performed in Ovid (databases were combined) for EMBASE (1974 to 15 October 2019), MEDLINE (1946 to 15 October 2019), PsycInfo (1906 to week 1 October 2019), and Joanna Briggs Institute EBP database (current to 15 October 2019).

Search terms (abstract, keywords, MeSH term, subject heading, title) were: primary care OR family practi* OR GP OR general practi* OR physician* OR primary health AND interview* OR advice OR consultation* OR promotion* OR counselling OR counselling AND motivation OR behaviour* change* OR behaviour* change* OR lifestyle change* AND physical activit* OR exercise* OR physiotherap* OR physical therap*. Where possible, the search was limited to humans. The search was repeated for the years 2019 and 2020 on 30 October 2020 for articles up to that date.

### Eligibility criteria

Included were peer-reviewed RCTs investigating behaviour change consultations promoting PA engagement in a primary care setting; studies whose outcome parameter include PA levels; studies with outcomes at the patient (that is, not clinician) level; articles reporting primary research studies in English, German, Italian, Spanish, French, or Dutch; and eligible studies retrieved through the reference lists of literature reviews.

Excluded were studies investigating interventions without reporting behaviour change consultations; studies examining consultations not pertaining to behaviour change and PA; and abstracts, protocols, editorials, discussion papers, and comments (unless relating to one of the included studies).

### Data management and screening

All records identified were imported into Mendeley, and duplicate records were removed. Title and abstracts were screened by one author to determine whether or not they met the eligibility criteria. The abstracts that did not meet the eligibility criteria were rejected and numbers were recorded. If the eligibility was uncertain, the article was retained, and its full text retrieved to determine eligibility.

Full-text articles for all candidate-eligible studies based on titles and abstracts were retrieved and assessed by two co-authors to determine eligibility. Any uncertainties concerning the appropriateness of reviews for inclusion were resolved through discussion with a third reviewer. Reasons for non-eligibility were recorded.

### Data extraction

Data from the selected articles were extracted by one author using a custom-designed form. Data extracted included author; year and country of publication; study characteristics, including design, and inclusion and exclusion criteria; participants; intervention characteristics, including frequency and duration; and outcome measures (primary and secondary) and effect of consultation on outcome measures (if possible).

### Assessment of risk of bias

Two authors appraised the quality of the included studies independently using the Cochrane Collaboration’s tool for assessing risk of bias in RCTs.^13^ Any disagreements were resolved through discussion with a third reviewer.

### Data analysis

For the first research objective, a descriptive analysis was completed to report who delivered the intervention (for example, primary care physician or practice nurse) and what type of behaviour change consultations were delivered. For the second research objective, two meta-analyses were completed for interventions, with a follow-up assessment at 6 and 12 months. These timepoints were chosen as they were the most commonly reported ones. As some studies included >1 PA measure (for example, minimum PA per week and metabolic equivalent of task (MET)-hours per week), the analysis was completed for results with the smallest effect size to provide a conservative estimate of the overall effect size. Effect sizes were based on standard mean differences for two samples. When >1 intervention was tested, effect sizes for each individual intervention were used in the meta-analyses. For the meta-analysis, effect sizes were weighted by sample size. For the third research objective, studies with moderate or large effect sizes were identified and their characteristics described.

## RESULTS

**Figure 1.**
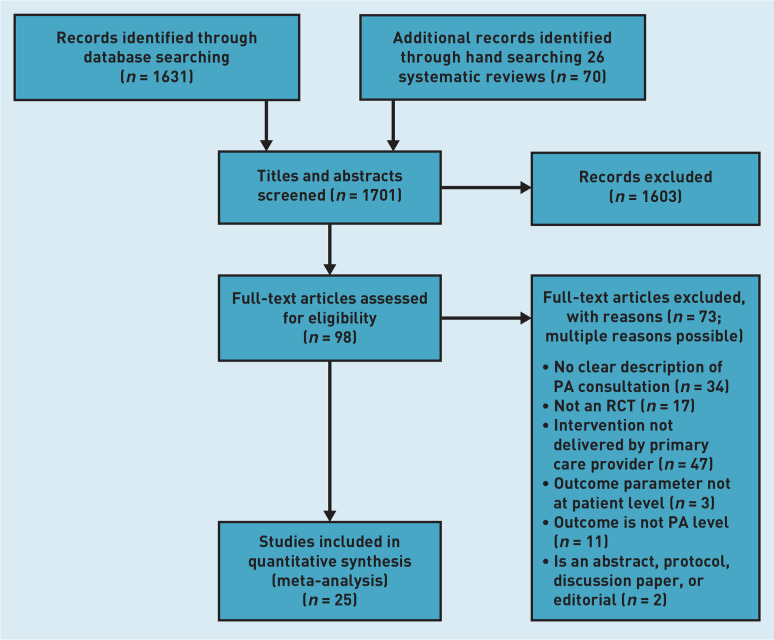
*PRISMA flow diagram.* *PA = physical activity. PRISMA = Preferred Reporting Items for Systematic Reviews and Meta-Analyses. RCT = randomised controlled trial.*

The screening process and reasons for exclusion of full-text articles are shown in Figure 1 as a Preferred Reporting Items for Systematic Reviews and Meta-Analyses (PRISMA) flow diagram.^14^ In total, 1701 articles were identified. Following titles and abstracts screening, 1604 articles were excluded. After full-text examination of the remaining 97 articles, 73 were excluded. The review included 25 articles.^15^^–^^39^ The characteristics of the included studies are reported in Supplementary Table S1. The studies were published between 1995 and 2020, with five articles each from the US,^15^^,^^16^^,^^29^^,^^32^^,^^33^ the Netherlands,^20^^,^^21^^,^^22^^,^^27^^,^^28^ and the UK,^25^^,^^30^^,^^34^^,^^35^^,^^36^ four from Australia^24^^,^^37^^–^^39^ two from Germany,^23^^,^^31^ and one each from Canada,^17^ Finland,^26^ Mexico,^18^ and Spain.^19^ The sample sizes ranged from 20 to 4317 participants.

### Quality appraisal

Most studies lacked details on randomisation and allocation concealment, as well as blinding of clinicians, researchers, and participants, though blinding was not possible in most study designs (Table 1). All studies except one,^15^ which did not show follow-up data, reported data loss. Fourteen studies used an intention-to-treat analysis;^17^^,^^19^^,^^21^^,^^24^^,^^26^^–^^28^^,^^30^^,^^31^^,^^34^^,^^35^^,^^36^^,^^38^^,^^39^ the other studies did not report how they approached the missing data in the analyses. Further bias might have been introduced in 18 studies either by not reporting fidelity data, or through low fidelity to the intervention.

**Table 1.** Risk of bias assessment, based on Higgins *et al* ^13^

**Author, year**	**Random sequence generation**	**Allocation concealment**	**Blinding of participants and personnel**	**Blinding of outcome assessment**	**Incomplete outcome data**	**Selective reporting**	**Other bias**
Ackermann, 2005^16^	+	+	+	+	+	+	+
Burton, 1995^15^	?	?	?	?	?	?	–
Christian, 2008^32^	+	+	+	?	+	+	?
Dubbert, 2002^29^	?	?	?	+	?	+	?
Galaviz, 2013^17^	?	?	–	?	+	+	+
Galaviz, 2017^18^	?	?	–	?	?	+	–
Goldstein, 1999^33^	?	?	–	?	?	+	–
Grandes, 2009^19^	+	–	–	+	+	+	?
Harris, 2015^36^	+	–	–	–	+	+	+
Harris, 2017a^38^	?	?	–	?	+	+	+
Harris, 2017b^34^	+	+	–	–	+	+	+
Jansink, 2013^20^	?	?	–	?	–	+	?
Jolly, 2018^30^	?	–	–	+	+	+	–
Kerse, 1999^39^	?	+	–	+	+	+	–
Koelewijn-van Loon, 2010^21^	?	?	–	+	+	+	?
Lakerveld, 2013^22^	+	+	–	+	–	+	?
Leonhardt, 2008^23^	?	?	–	?	+	+	+
Little, 2004^35^	?	?	?	+	+	+	?
Marshall, 2005^36^	?	?	–	+	+	+	–
McCallum, 2007^24^	+	+	–	+	+	+	–
Mehring, 2013^31^	+	+	–	–	+	+	–
Sims, 1999^25^	?	?	?	?	?	+	?
Valve, 2013^26^	+	?	–	?	+	+	?
Van der Weegen, 2015^27^	?	+	–	+	+	+	+
Westland, 2020^28^	+	+	–	–	+	+	–

*+ = low risk of bias.* – *= high risk of bias. ? = unclear risk of bias. Allocation concealment, as well as blinding of participants and clinicians delivering the intervention, was not possible in most study designs. All studies reporting follow-up data had reported data loss. If data loss was <15% and loss is even across groups or the loss was accounted for conservatively in data analysis (for example, intention to treat with replacing missing follow-up data with baseline values), the data loss was rated as low risk of bias. If adherence to the intervention was either not reported or <80% it was rated as high risk of bias in ‘other bias’.*

### Research objective 1

In nine studies, the intervention was delivered by primary care physicians,^15^^,^^17^^–^^19^^,^^24^^,^^32^^,^^33^^,^^37^^,^^39^ in 11 by practice nurses,^20^^–^^22^^,^^25^^–^^29^^,^^30^^,^^34^^,^^36^ and in five by both.^16^^,^^23^^,^^31^^,^^35^^,^^38^ Fifteen studies evaluated a PA intervention^16^^–^^19^^,^^23^^,^^25^^,^^27^^–^^29^^,^^31^^,^^33^^–^^37^ and 10 a lifestyle intervention.^15^^,^^20^^,^^21^^,^^22^^,^^24^^,^^26^^,^^30^^,^^32^^,^^39^^,^^40^ Three studies included a single behaviour change consultation as intervention,^16^^–^^18^ and 11 studies a baseline behaviour change consultation with follow-up visits or phone call.^19^^–^^28^^,^^36^ Three studies evaluated an intervention comprising telephone consultations,^29^^–^^31^ and six studies tested interventions that included behaviour consultation visits, as well as additional support mechanisms such as assessment of motivational readiness report, posters, or pedometers.^32^^–^^37^ Only two studies reported on the training for the practice staff but not on the implementation at the patient level,^38^^,^^39^ and one study included an intervention consisting of two physical examinations plus an optional behaviour change consultation.^15^

### Research objective 2

Though all interventions were consultation based, they still included a wide range of formats, types, and support mechanisms, with different follow-up periods. Therefore, the authors decided to complete the meta-analyses for studies with equal follow-up periods to enable a comparison of effects of the different interventions at a set timepoint. Due to a lack of detail in reporting, effect sizes could not be calculated for two of the studies.^22^^,^^29^

**Figure 2.**
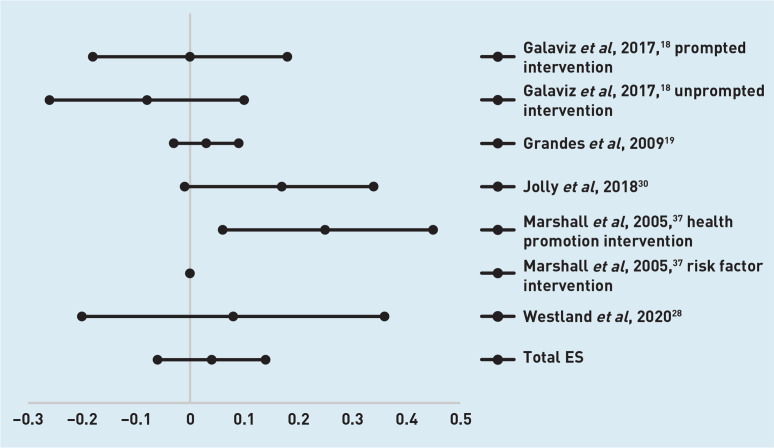
*Diagram of effect sizes (ES) and 95% confidence intervals of interventions with a follow-up assessment at 6 months.*

**Figure 3.**
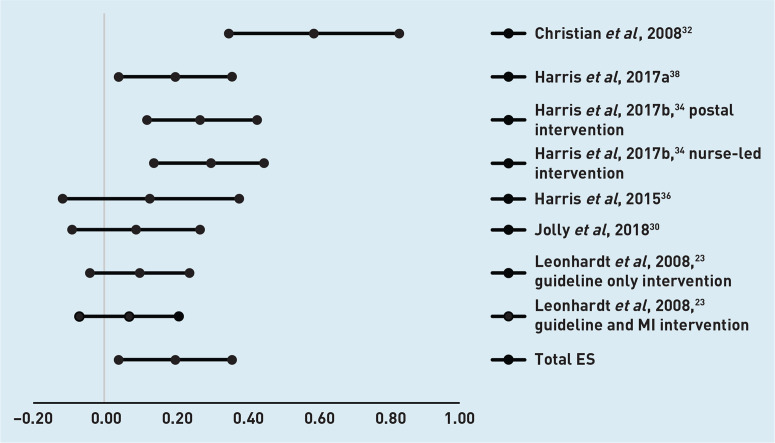
*Diagram of effect sizes (ES) and 95% confidence intervals of interventions with a follow-up assessment at 12 months.* *MI = motivational interviewing.*

For the five studies with eight interventions with a follow-up assessment at 6 months,^18^^,^^19^^,^^28^^,^^30^^,^^37^ the overall effect size was 0.04 (95% confidence interval [CI] = −0.06 to 0.14). Effect sizes and CIs are presented in Figure 2. Six studies with eight interventions had follow-up assessments at 12 months.^23^^,^^30^^,^^32^^,^^34^^,^^36^^,^^37^ The overall effect size was 0.19 (95% CI = 0.03 to 0.36) (Figure 3).

### Research objective 3

The effect of primary care counselling to increase PA levels was small for most studies, and better in studies designed to change behaviour over a longer period of time (12 months) than in studies with a shorter follow-up period (6 months). No further patterns identifying a successful intervention could be detected regarding specific intervention characteristics, such as counselling strategy, population, training of intervention staff, or theoretical underpinning of the intervention.

The only study that achieved a medium-effect size was by Christian *et al*,^32^ which included participants diagnosed with type 2 diabetes. The intervention was delivered by the primary care physician, and included three motivational interviewing sessions based on a personal report outlining the computer-assessed motivational readiness to increase PA and make dietary changes. The tailored report provided feedback to the participant, addressed behaviour change barriers, and listed two or three dietary and/or PA self-management goals that the participant had chosen as target behaviour. The participants were also given a 30-page planning guide with additional information about a healthy lifestyle. The physician received a summary of the participant’s report for the counselling visit to discuss goals.

## DISCUSSION

### Summary

Physical activity promotion may have a limited effect if restricted to primary care settings, despite different consultation approaches being used. Some studies included interventions investigating single counselling sessions; others had follow-up visits or telephone calls. Different support mechanisms, such as tailored reports, goal setting, or activity prescriptions were added, and a range of health psychology approaches were used as theoretical underpinning of the counselling element. There was no clearly superior counselling strategy, and only seven out of 24 interventions increased PA levels significantly more than their control interventions.

The effect sizes in the individual studies were generally small, and a meta-analysis of interventions with a 6- or 12-month follow-up period confirmed these findings. The difference in results between the meta-analyses with 6- and 12-month follow-up data also indicated that interventions developed for a long-term behaviour change (here 12 months) might be more effective that those developed for a shorter-term follow-up. Because of the lack of reporting on details regarding the content of the counselling sessions, it remains unclear if the prospect of a 12-month follow-up affected the counselling approach.

The only study including an intervention that showed a moderate effect size was by Christian *et al*.^32^ Their intervention design included characteristics (for example, detailed assessment of readiness and goal setting) that have been shown to support behaviour change in overweight and obese people.^10^ The findings of the review by Samdal *et al* showed that goal setting and self-monitoring were significantly associated with a positive intervention effect both in the short and long term.^10^ This would suggest that interventions to increase PA might work better for certain subgroups, as the sample of the Christian *et al*^32^ study included people with type 2 diabetes.

### Strengths and limitations

This systematic literature review is the first to investigate effect sizes of PA promotion counselling in primary care settings. Though only interventions based on counselling were included, the review examined different approaches without external support that might not be available for primary care patients. The meta-analysis contained studies based on the length of the follow-up interval (6 and 12 months), but these included a wide range of intervention characteristics.

Overall, the quality of the included studies was acceptable, though some studies did not report sufficient details on randomisation, blinding of participants, and intervention deliverers (primary care physicians and praxis nurses). Fidelity reporting was lacking in many studies and it was therefore not always clear whether the small effect was due to the intervention itself, or whether the intervention had not been implemented as intended. Process evaluation and adherence reporting are an essential part of a RCT.^40^ Without these, the findings lack the required context to conclude whether the intervention itself was inefficient, or whether the implementation of the intervention was unsuccessful. Any future RCTs should include a well-designed process evaluation that follows Medical Research Council guidelines.^40^ Furthermore, because of different followup periods, not all studies could be included in the meta-analyses and there were not enough studies to compare the effect of different counselling approaches. Another limitation was the number of literature databases used for the search; this was due to time and resource constraints. Though the literature databases used in this review included large scientific databases for medical research, additional articles might have been identified by searching a wider range of databases.

### Comparison with existing literature

The review excluded interventions that contained elements not delivered in a primary care context, such as exercise classes, external support (for example, from psychologists or exercise trainers), and/ or community groups. In addition, further motivation support strategies such as fitness trackers can support self-monitoring and exercise adherence.^41^^,^^42^ Linking primary care counselling with additional elements of PA support might lead to larger effects on PA behaviour. A more comprehensive approach to behaviour change, with multiple support mechanisms, would also better reflect the behaviour change wheel by Michie *et al*,^9^ which suggests that a comprehensive behaviour change support system rather than one source is required to support the person to change their behaviour. Three components, motivation (brain processes that energise and direct behaviour), capability (a person’s capacity to engage in the targeted activity), and opportunity (external factors that make the behaviour possible or prompt it), are required to achieve a positive behaviour change.^9^ A successful intervention should focus on all three components to provide a supporting context for the individual to adopt a healthy lifestyle.

### Implications for research and practice

The findings indicate that counselling to promote PA in primary care has a limited effect on patients’ behaviour. Strategies to increase PA levels should include a more comprehensive approach, with multiple mechanisms to support motivation, capability, and opportunity, rather than a single point of encouragement for behaviour change in primary care. Future interventions should use a comprehensive approach as outlined in Michie’s behaviour change wheel^9^ to develop interventions and report these in sufficient detail to allow replication of the research. The RCTs testing the interventions need to include a process evaluation to assess the implementation of the intervention and to clarify causal mechanisms and context factors. The combined information from the intervention development reporting and the results of the RCT, as well as the process evaluation, could then enable a detailed analysis of which intervention components enable behaviour change mechanisms.
